# Dysbiosis in the Rhizosphere Microbiome of Standing Dead Korean Fir (*Abies koreana*)

**DOI:** 10.3390/plants11070990

**Published:** 2022-04-05

**Authors:** Gil Han, Mohamed Mannaa, Hyoseong Jeon, Hyejung Jung, Jin-Cheol Kim, Ae Ran Park, Young-Su Seo

**Affiliations:** 1 Department of Integrated Biological Science, Pusan National University, Busan 46241, Korea; croone@pusan.ac.kr (G.H.); mannaa@cu.edu.eg (M.M.); jhj4059@pusan.ac.kr (H.J.); 2 Department of Plant Pathology, Cairo University, Giza 12613, Egypt; 3 Division of Applied Bioscience and Biotechnology, Chonnam National University, Gwangju 61186, Korea; gytjd9633@naver.com (H.J.); kjinc@chonnam.ac.kr (J.-C.K.); arpark9@naver.com (A.R.P.)

**Keywords:** endangered species, metagenomic analysis, Korean fir, rhizosphere

## Abstract

The Korean fir *(Abies koreana),* a native coniferous tree species mainly found on Mt. Halla in Jeju, South Korea, is suffering from continuous population decline and has been declared an endangered species. Research efforts have focused on the possible abiotic causes behind this worrying decline. However, the potential link between tree vitality and the rhizosphere microbiome remains unclear. In this study, a comparative metagenomic 16S rRNA sequence analysis was used to investigate the composition of the rhizosphere microbiota of samples collected from healthy and die-back-affected trees on Mt. Halla. The results indicated a significant reduction in the richness and diversity of microbiota in the rhizosphere of die-back-affected trees. Moreover, the relative abundance of Proteobacteria, Actinobacteria, and Bacteroidetes were significantly higher in healthy trees than in standing dead trees. Many bacterial genera were significantly more abundant in the rhizosphere of healthy trees, including those known for promoting plant growth and tolerance to biotic and abiotic stresses (e.g., *Bradyrhizobium*, *Rhizomicrobium*, *Caulobacter*, *Nitrosospira*, *Rhizobacter*, *Paraburkholderia*, *Rhizobium*, *Devosia*, *Caballeronia*, *Niveispirillum*, *Dyella*, *Herbaspirillum*, *Frankia*, *Streptomyces*, *Actinoallomurus*, *Lysobacter*, *Luteibacter*, *Mucilaginibacter*, and *Variovorax*). To our knowledge, this is the first report on rhizosphere bacterial microbiome dysbiosis in die-back-affected Korean fir trees, suggesting that the influence of rhizosphere microbiota should be considered to save this endangered species by investigating possible intervention strategies in future work.

## 1. Introduction

The Korean fir *(Abies koreana),* first reported in 1920, is an endemic subalpine species located on four tall mountains in the southern area of South Korea: Mt. Halla, Mt. Togyu (Deojyu), Mt. Chiri (Jiri), and Mt. Gaya [1]. The largest number of these trees are found on Mt. Halla, a volcanic mountain with the highest peak (~1950 m altitude above sea level) located in the center of the largest island in the Korean peninsula, Jeju Island [2,3]. The Korean fir tree was declared an endangered species (https://www.iucnredlist.org/species/31244/9618913, accessed on 1 February 2022) by the International Union of Conservation of Nature (IUCN) because of its continuous decline in numbers since the 1980s [4].

Researchers are exploring the reasons behind the worrying decline in Korean fir trees. Multiple contributing factors have been hypothesized; the main players, including heat stress, increased precipitation rates, reduced sunshine durations, heavy winter snow, strong winds, moisture imbalance, and vegetation changes in the area, are associated with climate change [5,6,7,8,9]. Studying tree mortality rates in relation to the degree of slope and solar radiation has shown that poor drainage networks and excess soil moisture may contribute to the observed decline [10]. Although several studies have established strong links between tree population decline and several factors, the exact causes of the decline remain unclear. Furthermore, the rate of decline is expected to increase with global warming [6]. The vulnerability of trees to strong winds and harsh, changing climates have also been attributed to the fact that these trees are situated on a lava floor—Mt. Halla is volcanic in nature—with relatively shallow soil that may be insufficient to support the root systems of big trees [11].

Extensive research efforts have been devoted to investigating the roles of abiotic factors in the status of endangered Korean fir trees. Little is known about biotic factors and their potential contribution to the health status of these trees. Currently, plants and a large number of associated microbes are considered holobionts, which live in cooperative mutual interactions that are indispensable for their stability, fitness, and survival [12]. Accumulating knowledge about the plant microbiome based on recent advances in analytical tools [13] has illustrated its significance in plant growth, with diverse forms of interactions within their mutualistic to commensalistic and pathogenic relationships. In particular, the soil and rhizosphere (i.e., the part of the soil under the influence of root exudates) microbiome represent the most well-known biological diversity reservoirs that play key roles in sustaining plant growth by facilitating metabolic capabilities and nutrient uptake and enhancing tolerance to biotic and abiotic stresses [14]. Due to a large number of detected species, cells, and the rhizosphere genetic potential contributing and interacting with plants, it is considered the plant’s second genome [15]. Plants rely on their second genome for their development and health, as they devote a considerable portion of their photosynthetically fixed carbon to recruit, shape, feed, and manage their associated microbes [15,16]. Hence, understanding the processes that shape the rhizosphere microbiome and deciphering the composition, roles, and interplay between the plant-rhizosphere microbiome would be critical for sustaining plant growth and productivity.

The association between plants and the rhizosphere microbiome is even more specific and substantial in forest trees. Forest trees establish more stable relationships with the underground microbial community because of their long-living nature, persistent deep root system, and large-scale deposition of root exudates as an energy source, a driving factor for shaping the microbial structure [17]. As has been the case with human gut microbiome research and the current conclusions about their influence on human health, the tree rhizosphere microbiome may perfectly fit into the same context, as it is also among the richest microbial ecosystems. Thus, the rhizosphere microbiome can be viewed as the “tree gut microbiome,” where dysbiosis can have a profound influence on plant health. Even a small change in underground microbial diversity can have a significant impact on ecosystems [18]. Based on this observation, we propose that changes in the rhizosphere microbiome may be linked as a cause or potential ecophysiological response to the decline in the number of Korean fir trees.

Previous studies on other forest trees in endangered situations due to climate change-related factors, such as cork oak forests, have indicated the importance of the rhizosphere microbiome as a key component within the ecosystem contributing to its sustainability, as rhizosphere microbiome degradation was found to accelerate the decline process [19]. A previous study on Chinese fir trees reported varying rhizosphere microbiota diversity, richness, and composition largely attributed to environmental conditions, including soil properties and precipitation rates [20]. Hence, the rhizosphere microbiome should be considered as a major aspect in studying forest ecosystems and among the factors contributing to the status of trees. A previous recent study has investigated the community structure of higher fungi between dead and living Korean fir trees and indicated the significant differences in the composition [21]. However, the bacterial rhizosphere composition and links to tree status remain unclear.

Given the importance of the rhizosphere microbiome to forest sustainability and the health status and stress tolerance of trees, it is surprising that there has not been any previous study investigating the bacterial rhizosphere microbiome of Korean fir trees and their possible links to tree sustainability as an ecophysiological response or contributing factor to tree population decline. Therefore, this study aimed to utilize high-throughput sequencing-based 16S rRNA metabarcoding to analyze the bacterial rhizosphere microbiome of Korean fir trees and unravel possible differences in microbial diversity, richness, and composition between healthy and decline-affected dead trees. Deciphering the differences between healthy and dead tree rhizosphere microbiomes may be useful for determining the microbial taxa that can be exploited for improving the growth, health, and sustainability of Korean fir trees in future endeavors.

## 2. Results

### 2.1. Metagenomic 16S rRNA Sequencing Results

A high-throughput metagenomic 16S rRNA sequencing of the 30 rhizosphere samples from healthy and dead Korean fir trees resulted in 2,810,535 total reads with an average of 93,685 reads per sample. Details of the obtained raw sequence read for each sample, including the total base count, total reads, GC%, Q20%, and Q30%, are shown in Table S1. Following sequence pre-processing and clustering using CD-HIT-OTU to remove low-quality, short reads, and chimera, the total number of clean non-chimeric reads was 417,062 with an average of 13,902, a minimum of 8638, and a maximum of 18,315 reads per sample.

### 2.2. Rhizosphere Microbiome Diversity and Richness in Healthy and Dead Korean Fir Trees

**Figure 1:**
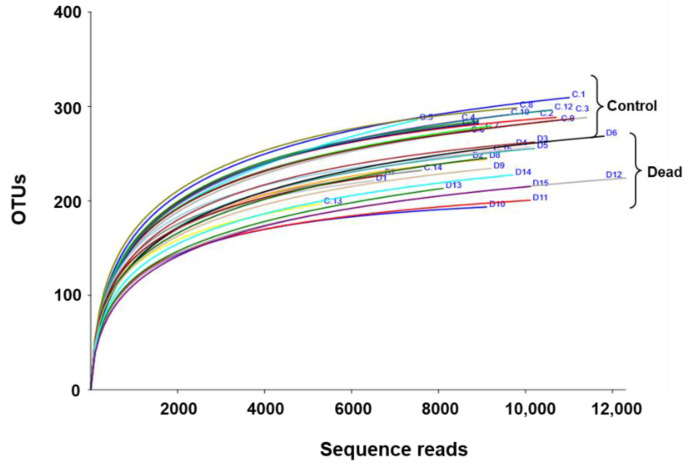
Rarefaction curves of the metagenomic 16S rRNA sequence reads from the Korean fir tree rhizosphere plotted against the assigned operational taxonomic units (OTUs). The analysis revealed sufficient sequencing depth to reflect the major components of the microbiome composition. Generally, a higher number of OTUs was observed in the control samples compared to the standing dead trees.

A sequencing depth that can satisfactorily represent the main components of the rhizosphere microbiome in the collected samples was confirmed via rarefaction analysis. The results showed the number of individual operational taxonomic units (OTUs) identified in a given number of reads, indicating that the OTUs reached a near plateau at approximately 4000 reads (Figure 1). A noticeably higher number of OTUs was also observed in the control samples of healthy Korean fir trees than in those that died. 

**Figure 2:**
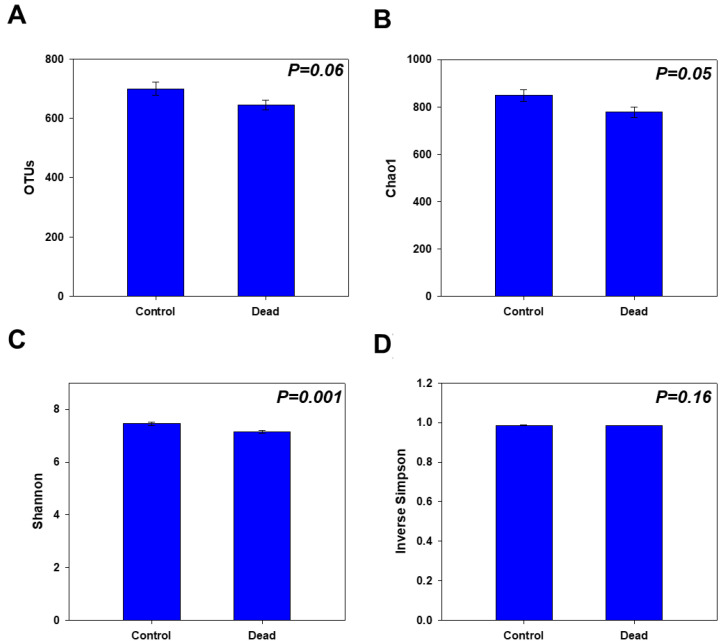
Bar graphs of the alpha diversity measures of the control healthy and standing dead Korean fir tree rhizosphere microbiomes. The graphs show the (**A**) number of operational taxonomic units (OTUs), (**B**) Chao1 indices, (**C**) Shannon diversity indices, and (**D**) inverse Simpson indices. Bars represent the means ± standard errors (*n* = 15). The calculated *p*-values from Student’s *t*-test are shown on the graphs.

The alpha diversity measures further confirmed the observed differences between the two groups, as the healthy trees showed noticeably higher diversity and richness in their rhizosphere microbial community than the standing dead trees. The number of OTUs, and the Chao1 and Shannon diversity indices were significantly higher (*p* < 0.05) in the control samples of healthy trees than in those of dead trees (Figure 2A–C). However, the inverse Simpson measure did not show a significant difference between the groups (Figure 2D). 

**Figure 3:**
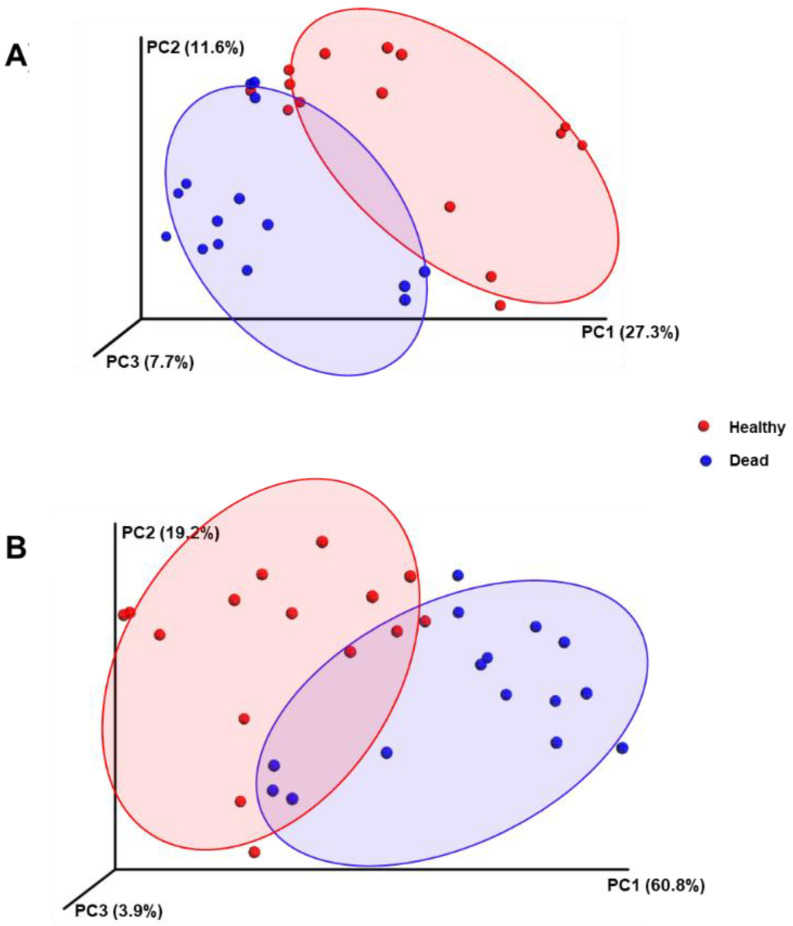
Three-dimensional graphs representing the beta-diversity of the Korean fir tree rhizosphere microbiome samples expressed as a principal coordinate analysis (PCoA) of the (**A**) unweighted UniFrac and (**B**) weighted UniFrac distances. A clear distinction was observed as the healthy control samples were clustered separately from the standing dead samples.

When the beta diversities between the tested groups were considered, a noticeable distinction was observed in the principal coordinate analysis (PCoA), which was based on the weighted and unweighted UniFrac, as the samples collected from the rhizosphere of the control healthy trees were grouped in a relatively distinct cluster from the dead tree samples (Figure 3A,B). This result indicates a difference in the rhizosphere microbial composition between the control and dead Korean fir trees when the microbial types and abundances were considered.

### 2.3. Taxonomic Characterisation and Comparative Analysis of the Rhizosphere Microbiome Structure between Healthy and Dead Korean Fir Trees

**Figure 4:**
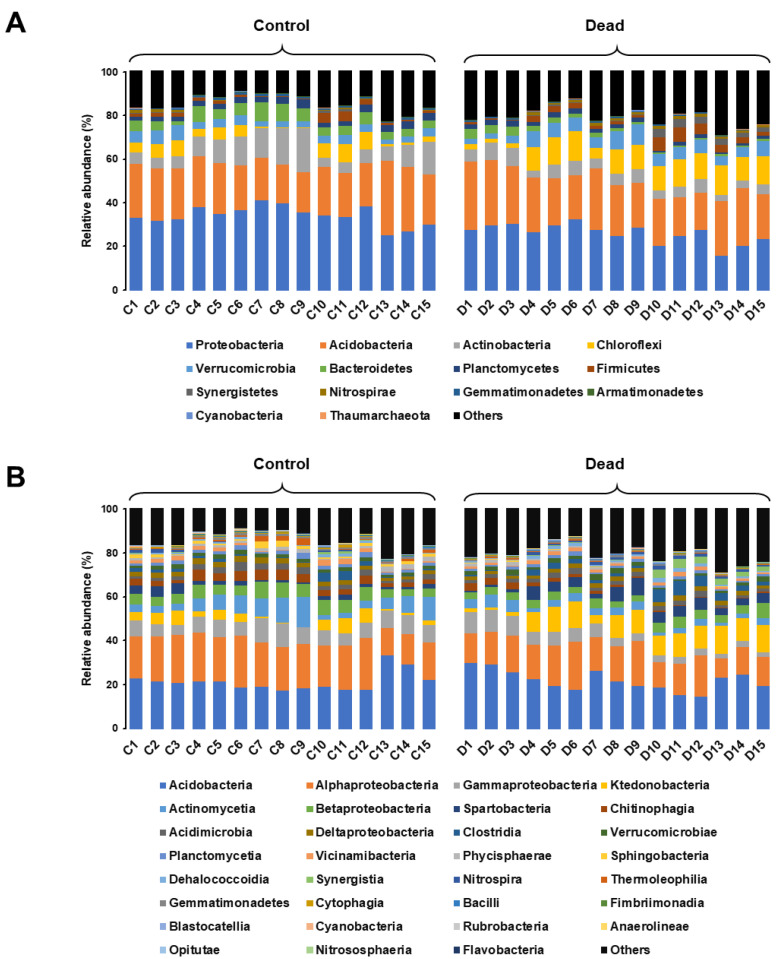
Stacked bar graphs representing the microbiota structure of Korean fir tree rhizospheres at (**A**) the phylum and (**B**) the class levels. The samples are representative of the healthy control and standing dead trees.

The stacked bar graphs in Figure 4A,B show the relative abundances of the rhizosphere microbial composition in healthy and dead Korean fir trees at the phylum and class levels, respectively. Generally, at the phylum level, the rhizosphere microbial community of the control healthy trees was dominated by Proteobacteria, Acidobacteria, Actinobacteria, and Bacteroidetes, accounting for an average of 71.48% of the total identified taxa. The dead tree rhizosphere microbiome was dominated by Proteobacteria, Acidobacteria, Chloroflexi, and Verrucomicrobia, accounting for an average of 64.54% of the total identified taxa. The control group contained significantly higher (*p* < 0.05) relative abundances of Proteobacteria, Actinobacteria, Bacteroidetes, Planctomycetes, and Gemmatimonadetes than the dead tree group, which had significantly higher relative abundances of Chloroflexi, Verrucomicrobia, Firmicutes, Synergistetes, and Nitrospirae (Figure 4A and Figure S1A). 

At the class level, the control group was dominated by Acidobacteria, Alphaproteobacteria, Gammaproteobacteria, Actinomycetia, and Betaproteobacteria, accounting for an average of 59.68% of the total identified taxa. In contrast, the dead tree rhizosphere microbiome group was dominated by Acidobacteria, Alphaproteobacteria, Ktedonobacteria, Gammaproteobacteria, and Spartobacteria, accounting for an average of 54.64% of the total identified taxa. The rhizosphere microbiome of the healthy trees group had a significantly higher (*p* < 0.05) relative abundance of Alphaproteobacteria, Gammaproteobacteria, Actinomycetia, Betaproteobacteria, Chitinophagia, Acidimicrobiia, Planctomycetia, Phycisphaerae, Sphingobacteriia, Thermoleophilia, Gemmatimonadetes, Cytophagia, Bacilli, Rubrobacteria, Opitutae, and Flavobacteriia compared to the dead tree group, which conversely had a significantly higher (*p* < 0.05) relative abundances of Ktedonobacteria, Spartobacteria, Clostridia, Synergistia, Nitrospira, Blastocatellia, and Nitrososphaeria (Figure 4B and Figure S1B). Bacterial taxa with significantly different relative abundances at the order and family levels between both groups were also reported (Figures S1C and S2).

**Figure 5:**
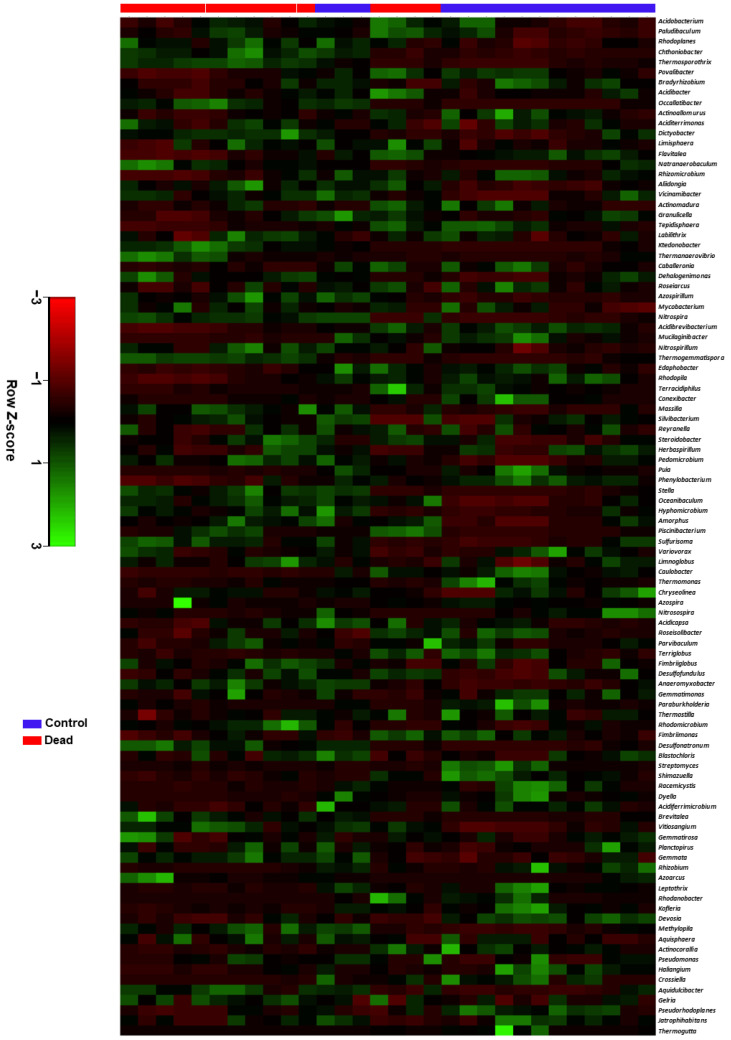
Heatmap based on the Euclidean distance measurement and the average linkage hierarchical clustering of the top 100 most abundant bacterial genera in the rhizosphere of Korean fir trees. A distinction was noticed between the control and the standing dead trees based on the differences in the abundance of the predominant bacterial genera. The Z-Score represents the relative abundance of the bacterial genera in each row after heatmap standardization.

The distinction between the healthy control and dead Korean fir tree rhizosphere microbiome compositions was further verified in a heatmap constructed based on Euclidean distance measurement. The average linkage hierarchical clustering for the top 100 represented bacterial genera in the microbial community of both groups is shown in Figure 5. The results showed a noticeable distinction between both groups as 12 out of the 15 samples from the control were clustered in a group separated from the dead trees group, where 11 out of the 15 samples were separated into another cluster.

### 2.4. Differential Occurrence of Bacterial Genera in the Rhizosphere Microbiota between Healthy and Dead Korean Fir Trees 

**Figure 6:**
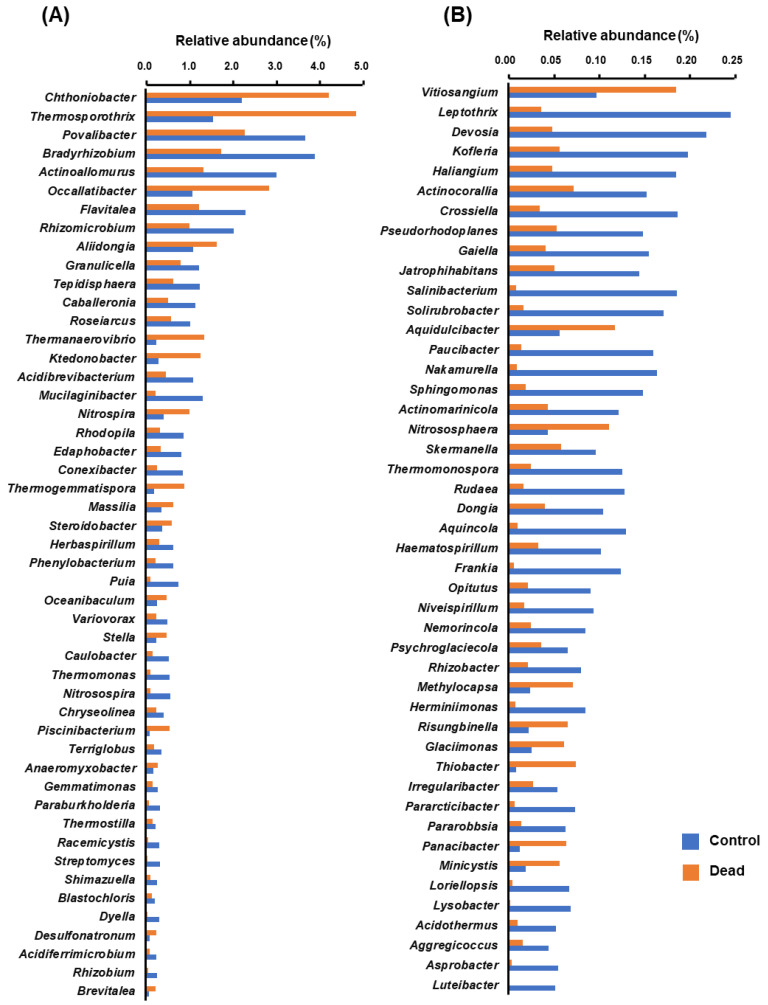
The bacterial genera with significantly higher (*p* < 0.05) relative abundances between healthy and standing dead Korean fir tree rhizosphere microbiome compared to the control. Bars represent the mean values (*n* = 15) of the relative abundance of the bacterial genera. (**A**) Bacterial genera represented relatively higher abundance levels (>0.28%). (**B**) Bacterial genera with relatively lower abundance levels (<0.28%).

In line with the previously stated results on the distinction between the control healthy and dead Korean fir tree rhizosphere microbiota, several bacterial genera were found to be present at significantly different levels (*p* < 0.05) of relative abundance between the healthy control and dead tree groups. The bar graphs in Figure 6 show the significantly different bacterial populations. For better visibility, the bacterial genera were divided into relatively highly represented genera (Figure 6A) and bacteria with relatively lower relative abundances (Figure 6B).

Among the detected OTUs, 70 bacterial genera were found to have significantly higher relative abundance levels (*p* < 0.05) in the rhizosphere of control healthy trees compared to dead trees. On the other hand, only 25 bacterial genera were found to have significantly higher relative abundances (*p* < 0.05) in the rhizosphere of dead trees than in healthy trees.

## 3. Discussion

This study was conducted on the endangered Korean fir tree species *A. koreana,* located mainly on Mt. Halla in Jeju, South Korea. Efforts are being made to understand the tree ecosystem, the different sources of biotic or abiotic stress (such as typhoons and periods of drought), and the exact reasons behind its observed population decline to determine the most efficient protective measures to conserve this species. Although there is a consensus from previous studies regarding a link between the decline in the population of Korean fir trees and climate change-related factors, a coherent explanation and the exact reasons for this decline remain unclear [8]. A wide variation was observed in the rate of population decline in the Korean fir tree at different locations on Mt. Halla, which indicates a possible specificity in the factors involved, which could be location-dependent. Ahn et al. [10] reported a possible correlation between the mortality rate of trees and topographic factors such as the degree of the slope, which could be linked to moisture-related stress in response to the degree of development of drainage networks. However, little is known about how these factors may be linked to the hidden world of underground rhizosphere microbial communities. 

In this study, the composition of the Korean fir tree rhizosphere microbiota was comparatively investigated between healthy and standing dead Korean fir trees using 16S rRNA gene metabarcoding. Soil and rhizosphere microbes play key roles in sustaining healthy plant growth and improving the adaptability, development, and productivity of forest trees [17]. The crucial roles of the rhizosphere microbiota include soil carbon dynamics, nutrient cycling, decomposition, antagonistic activity against phytopathogens, induction of plant resistance, and enhancement of plant tolerance to abiotic stress [22,23]. 

Our results indicated a significant decline in the diversity and richness of the rhizosphere microbiota in samples collected from dead trees compared to healthy ones, as seen in the total number of OTUs and the Chao1 and Shannon diversity indices. In agreement with these results, previous reports have highlighted the importance of microbial diversity as a critical factor in community fitness and competency [24,25]. The taxonomic diversity of the soil microbial community was found to be positively correlated with the diversity of the functional attributes of the community, as verified by Fierer et al. [18]. A more diverse and rich rhizosphere microbial community also results in enhanced microbiome functionality, such as disease suppression potential [26]. Hence, the significantly less diverse rhizosphere microbiome observed from the dead Korean fir trees in this study should be considered a potential factor that might contribute to the status of this endangered tree species. However, there are still many mysteries regarding this observed population decline. Determining the factors that cause the rhizosphere microbiome to lose diversity in die-back-affected trees, the changes in tree physiology in response to abiotic or biotic stresses, or the direct biological responses due to stress could elucidate the mechanisms underlying the decline in the Korean fir tree population.

Along with the statistical analysis of the microbial diversity and richness, the distinction between the microbial composition of the dead and healthy trees was further analyzed to reveal the rhizosphere microbiome composition of healthy trees. The results showed that healthy trees had significantly more Proteobacteria, Actinobacteria, and Bacteroidetes, while Firmicutes were significantly more abundant in the standing dead tree rhizosphere microbiome. Intriguingly, the same changes were observed in a previous study utilizing shotgun metagenomic approaches to characterize the microbial composition in unproductive soils and the changes that occurred upon their reclamation. The latter study reported that Firmicutes were more abundant in unproductive soils, while the reclamation of these soils (turning them into productive soils) resulted in a significant decrease in the abundance of Firmicutes and a significant increase in the abundance of Proteobacteria and Bacteroidetes, which was consistent with our results. These findings are evidence of the links between the changes in microbial taxa and the productivity or functionality of soils [27]. Moreover, in their ecological study of bacteria in a diverse array of soil and site characteristics, Fierer et al. [28] reported a significant negative correlation between the abundance of Acidobacteria and carbon mineralization rates. In contrast, a positive correlation was reported between the abundance of Betaproteobacteria and Bacteroidetes with carbon mineralization rates. Consistently, our study also showed that both Bacteroidetes and Betaproteobacteria were significantly more abundant in the rhizosphere of healthy trees than in the standing dead ones. 

Based on our results, Actinobacteria were among the significantly more abundant bacterial phyla in the rhizosphere of healthy trees compared to the standing dead trees, and are also among the most famous and ubiquitous bacterial groups known for their beneficial activities in cultivated soils. The plant-growth-promoting and other beneficial effects of Actinobacteria in the soil and rhizosphere are mainly attributed to diverse activities such as nitrogen fixation; phosphorous, potassium, and zinc solubilization; and the ability to produce diverse plant growth-promoting secondary metabolites, including phytohormones (e.g., indole-3-acetic acid, gibberellins, and cytokinins), antibiotics (e.g., kanosamine, neomycin A, phenazine-1-carboxylic acid, pyocyanin, and pyrrolnitrin), lytic enzymes (e.g., chitinase and protease), and siderophores [29,30]. These beneficial properties suggest that actinomycetes are good candidates for improving the functionality and bioremediation of soils [31].

In addition to promoting plant growth and suppressing disease, beneficial rhizosphere microbes also facilitate plant growth and tolerance to abiotic stress. This is particularly important with respect to our study, considering that the declining population of Korean fir trees was consistently attributed to abiotic factors that are being recently amplified in effect and increasing in frequency, apparently due to climate change [8]. Beneficial plant-associated microbes might contribute to enhancing plant endurance to different abiotic stresses, such as drought, waterlogging, and temperature, by their potential to stimulate priming conditions through a cascade of signaling pathways. This leads to preconditioning the plants to combat stress more strongly, quickly, and effectively via particular physiological alterations and biochemical makeup adjustments by controlling the transcription and regulation of stress-responsive genes [32,33,34]. Recently, metabolomics and other omics-based studies have attempted to elucidate the mechanisms underlying the action of microbes in inducing plant priming to alleviate abiotic stress, exploring the potential use of biostimulant-based strategies for improving plant health and resilience in the context of climate change [34,35,36]. Actinomycetes, as explained above, were represented in significantly higher abundance in our study in the rhizosphere samples collected from healthy trees compared to dead ones and are among the main microbial taxa reported to be involved in enhancing plant endurance to abiotic stress [37]. These actions of Actinomycetes could be attributed to their peculiar tolerance to harsh environments combined with their diverse abilities to improve plant health under stress using mechanisms such as manipulation of phytohormones, plant roots, and cell wall modification to avoid oxidative damage and assist in osmoregulation [34]. Several members of Actinomyces, such as those belonging to the genus *Streptomyces*, are bioinoculants that improve drought tolerance in plants [38].

The results of this study also showed that several bacterial genera were significantly more abundant in the rhizosphere of healthy trees than in dead trees. Among these bacterial genera, as evidenced in previous studies, several species belonging to these groups are known as plant-growth-promoting and beneficial rhizosphere microbes, including root nodulators and free-living nitrogen-fixing bacteria (e.g., *Bradyrhizobium, Rhizomicrobium, Caulobacter, Nitrosospira, Rhizobacter, Paraburkholderia, Rhizobium, Devosia, Caballeronia, Niveispirillum, Dyella,* and *Herbaspirillum*), beneficial Actinomycetes (e.g., *Frankia, Streptomyces,* and *Actinoallomurus*), and other beneficial metabolite-, phytohormone-, and siderophore-producing phytopathogen-antagonistic bacteria (e.g., *Lysobacter, Luteibacter, Mucilaginibacter,* and *Variovorax*) [39,40,41,42,43,44,45,46,47,48,49,50,51,52,53,54]. 

The beneficial roles of these bacterial genera are attributed to their ability to promote and support healthy plant growth by facilitating nutrient availability and acquisition, their ability to suppress phytopathogens, and support plant growth under abiotic stress [52,53,54,55]. Interestingly, a strain of *Variovorax* was shown in a previous study to have the ability to counteract the effect of drought stress in plants cultivated in dry soils. A species of *Variovorax* was found to enzymatically degrade the precursor of ethylene (a phytohormone produced in drought-affected plants and is responsible for the inhibition of plant growth) in plants and amplify the concentration of xylem abscisic acid. These bacterial activities enhance the efficiency of water use and promote the growth of drought-affected plants via local and systemic hormone signaling [55].

The bacterial genera in declining trees were also investigated for possible significant activities. Although there were no phytopathogens, specific members such as *Massilia*, were found at a significantly higher relative abundance in the rhizosphere samples collected from the declining trees. Members of the *Massilia* genus were shown in a previous study to have a reduced population in suppressive soils of fungal pathogens and to be negatively correlated with important plant-growth-promoting microbes such as *Rhizobium, Paenibacillus*, and *Streptomyces* [56]. Fungal pathogens are known to be important factors affecting the forest trees’ health status and a clear distinction in the composition was previously reported in a comparative study [21]. A previous study also reported the effect of soil-borne fungi on the overwintering survival of *A. koreana* seeds. The latter study indicated that certain fungal species, especially *Racodium ryanum*, had a detrimental effect on the seed germination ability [57]. It is, therefore, necessary to consider fungal integration with bacterial communities in future investigations of the biotic factors involved in the tree decline.

## 4. Materials and Methods

### 4.1. Study Site and Tree Selection 

**Figure 7:**
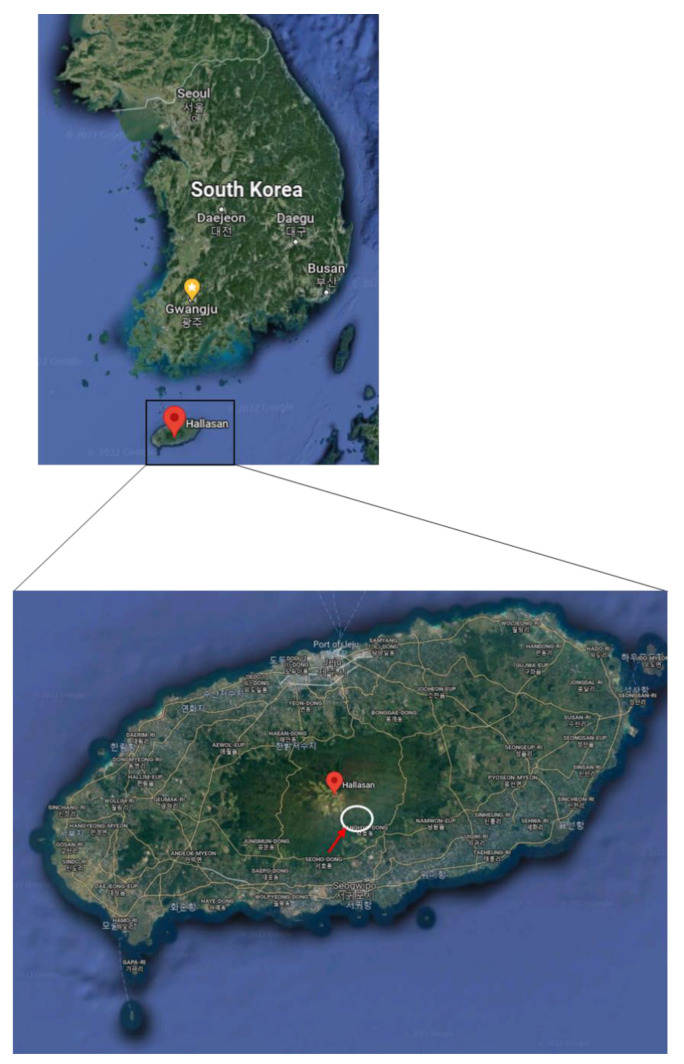
The sampling location of the Korean fir trees on Mt. Halla at an altitude of approximately 1700–1800 m above sea level.

The Korean fir tree *Abies koreana* used in this study was selected from Mt. Halla on Jeju Island, South Korea. Samples were collected from endangered trees located on the southern slope near the Mt. Halla center peak (33°21′ N, 126°32′ E) at an altitude of 1700–1800 m above sea level (Figure 7). Samples were collected from healthy and die-back-affected dead trees in the same sites and only closely attached soils were used to eliminate the effect of location-related differences in the pedoclimatic conditions. Since Mt. Halla is a volcanic mountain, the soil of that location is mostly volcanic ash soil. The annual average temperature of the region is 15 to 16 °C with average annual precipitation of 1500–1600 mm.

**Figure 8:**
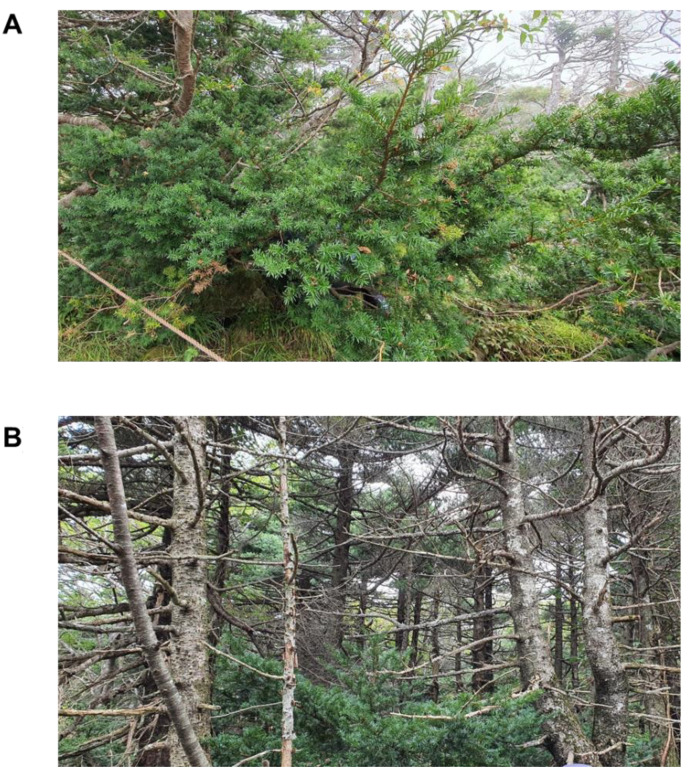
Images of Korean fir trees (*Abies koreana*) used for sample collection in this study. (**A**) Healthy looking stable trees and (**B**) die-back-affected standing dead trees.

The trees were divided into two groups: control healthy trees (showing normal vegetative growth and green needles) and standing die-back-affected dead trees (Figure 8).

### 4.2. Sample Collection and Metagenomic DNA Extraction 

Samples were collected from the roots and adjacent soils of healthy controls (*n* = 15) or standing dead trees (*n* = 15). Non-attached soils (bulk soils) were removed from the collected samples before processing, and only the rhizosphere soils were used for further analysis. For sample preparation, approximately 5 g of the collected sample was suspended in 20 mL of sterile distilled water and vortexed for 5 min. Excess water was removed from the suspension via centrifugation at 10,000× *g* for 15 min. From each sample, 250 mg of the collected pellets were used for metagenomic DNA extraction using a PowerSoil^®^ DNA Isolation Kit (Qiagen, Hilden, Germany; previously Mo Bio Laboratories), according to the manufacturer’s instructions. The quality and concentration of the obtained rhizosphere metagenomic DNA was evaluated using agarose gel electrophoresis and a NanoDrop2000 spectrophotometer (Thermo Fisher Scientific, Wilmington, NC, USA). Samples with satisfactory concentration and quality were stored at −20 °C in a Tris-EDTA buffer solution until use.

### 4.3. Metagenomic 16S rRNA Amplification, Sequencing, and Microbiome Analysis 

The hypervariable V3 and V4 regions of the 16S rRNA sequences were used for metagenomic analysis via high-throughput next-generation sequencing following amplification, according to the Herculase II fusion DNA polymerase Nextera XT Index Kit V2 protocol in the Illumina^®^ MiSeq^®^ platform at Macrogen (Seoul, South Korea), using the following primer pair: 

(F) 5′-TCGTCGGCAGCGTCAGATGTGTATAAGAGACAGCCTACGGGNGGCWGCAG-3′, and

(R) 5′-GTCTCGTGGGCTCGGAGATGTGTATAAGAGACAGGACTACHVGGGTATCTAATCC-3′.

The obtained raw paired-end sequences were merged using fast length adjustment of short reads (FLASH; http://ccb.jhu.edu/software/FLASH/, accessed on 1 February 2022) [58]. The adaptors and short reads were trimmed and the merged sequences were purified. The CD-HIT-OTU-MiSeq and UCLUST algorithms were also used to cluster and annotate the qualified pure sequences into their operational taxonomic units (OTUs), setting 97% as the cut-off value according to the Greengenes database [59,60,61]. Rhizosphere microbiome analysis was conducted following the pipeline of quantitative insights into microbial ecology version 2 (QIIME2), resulting in the analysis of the alpha and beta diversity statistics and the taxonomic assignment of the detected OTUs [62]. The obtained sequences were deposited in the National Center for Biotechnology Information database as a sequence read archive under BioProject ID PRJNA799867.

### 4.4. Statistical and Data Analysis 

Korean fir tree rhizosphere 16S rRNA microbiome analysis, including diversity statistics, was performed using the QIIME2 scripts and R software (version 3.1.3). The PAlaeontological STatistics software package (PAST) version 3.23 was used for the rarefaction curves of the obtained sequences to the detected OTUs [63]. The beta diversity between the control and dead trees was visualized based on weighted and unweighted UniFrac distance estimations. A heatmap was constructed using Euclidean distance measurements and average linkage hierarchical clustering between the samples. The student’s *t*-test was used to compare the differences between the two groups regarding alpha statistics and the differentially existing bacterial taxa at different taxonomic levels. A *p*-value < 0.05 was considered statistically significant. 

## 5. Conclusions

To the best of our knowledge, the present study was the first to investigate the community structure of the rhizosphere bacterial microbiota between healthy and standing dead Korean fir trees on Mt. Halla, South Korea. The results showed a clear distinction between the microbial composition in both groups. In particular, there was a relative loss in diversity and richness and a significant increase in the relative abundance of several microbial taxa known for their beneficial roles in the soil and rhizosphere and for promoting plant growth. Several bacterial groups, such as those belonging to the genera *Streptomyces* and *Variovorax*, have been shown to support plant growth under drought stress and have been suggested as biostimulants for enhancing plant drought tolerance and alleviating stress. 

The observed loss in microbial diversity and reduction in the abundance of beneficial microbes could be viewed as dysbiosis that correlates with the vulnerability of a tree to stress. Therefore, based on our results regarding the clear distinction in the microbial profiles and the annotated beneficial functional attributes in the rhizosphere microbiome of healthy trees, we propose considering the underground rhizosphere microbiome among the factors investigated to contribute to the worrying decline in Korean fir tree populations.

Future studies should investigate factors affecting and shaping the rhizosphere microbial community and the links between the observed changes in the microbial composition and their direct interaction with tree physiology, which might contribute to the health status of trees. Furthermore, future studies should also consider a more comprehensive large-scale approach including the fungal and bacterial microbiomes of healthy and declining trees and taking into account the soil physicochemical and pedoclimatic conditions.

The emerging concept of microbiome engineering and advanced approaches for manipulating the microbiome should be considered to save Korean fir trees and their ecosystems. To achieve this goal, bio-inoculating with candidate microbes, the use of diverse microbial consortia, plant-mediated engineering to recruit beneficial microbes, and the use of innovative tools (such as CRISPR gene editing techniques for microbiome engineering) should be explored further.

## Data Availability

The obtained sequences were deposited in the National Center for Biotechnology Information database as a sequence read archive under BioProject ID PRJNA799867.

## Supplementary Materials

The following supporting information can be downloaded at: https://www.mdpi.com/article/10.3390/plants11070990/s1. Figure S1. Bar graphs representing the relative abundance of the significantly different (*p* < 0.05) bacterial taxa at the (A) phylum, (B) class, and (C) order levels in the rhizosphere microbiome of healthy and standing dead Korean fir trees. Figure S2. The relative abundance of significantly different bacterial families (*p* < 0.05) between the standing dead Korean fir tree rhizosphere microbiomes and healthy controls. Bars represent the mean values (*n* = 15) of the relative abundance of the bacterial families. Table S1. The high throughput sequencing results represent the total bases, read count, GC%, Q20%, and Q30% of samples collected from healthy or standing dead Korean fir tree rhizosphere microbiomes.
