# Supplementary material for: Dysbiosis in the Rhizosphere Microbiome of Standing Dead Korean Fir (Abies koreana)
Source: Plants (Basel). 2022 Apr 5;11(7):990. doi: 10.3390/plants11070990 (PMC9002731; doi:10.3390/plants11070990)
Supplement: Supplementary file 1 [file plants-11-00990-s001.zip › plants-1649996-supplementary.pdf]

Table S1. Results of the high throughput metagenomics sequencing representing the total bases, read count, GC%, Q20% and Q30% of samples collected from the Korean fir tree rhizosphere

Sample	Total Bases	Read Count	GC (%)	Q20 (%)	Q30 (%)
C1	47653913	105754	55.84	98.84	95.84
C2	44764009	99421	56.09	98.75	95.61
C3	44629428	99188	56.47	98.79	95.76
C4	40591665	90141	55.61	98.7	95.49
C5	30944226	68766	56.04	98.4	94.38
C6	37100391	82460	55.88	98.63	95.23
C7	43529212	96508	55.1	98.71	95.51
C8	46174042	102288	55.1	98.86	95.98
C9	45942291	102013	55.63	98.73	95.6
C10	43990849	97603	56.29	98.68	95.43
C11	40564049	90079	56.28	98.78	95.67
C12	44827919	99651	56.04	98.77	95.7
C13	31503727	70282	55.86	98.56	94.98
C14	38704943	86243	55.94	98.92	96.08
C15	46170731	102820	56.02	98.74	95.55
D1	42597278	94828	56.11	98.57	95.07
D2	46899896	104451	56.14	98.78	95.71
D3	52895905	117673	55.98	98.78	95.73
D4	41934426	93201	56.51	98.66	95.27
D5	37474830	83399	56.53	98.55	94.93
D6	46136759	102803	56.66	98.79	95.72
D7	35033856	77879	56.49	98.8	95.71
D8	44968740	99991	56.85	98.61	95.18
D9	46782102	104174	56.94	98.54	94.87
D10	40835059	90665	57.28	98.61	95.08
D11	38872779	86380	57.23	98.53	94.88
D12	43211493	96144	57.25	98.7	95.41
D13	39539709	87963	57.21	98.75	95.51
D14	43052491	95804	57.02	98.78	95.71
D15	36842519	81963	56.99	98.75	95.57

C, healthy control; D, standing dead trees.

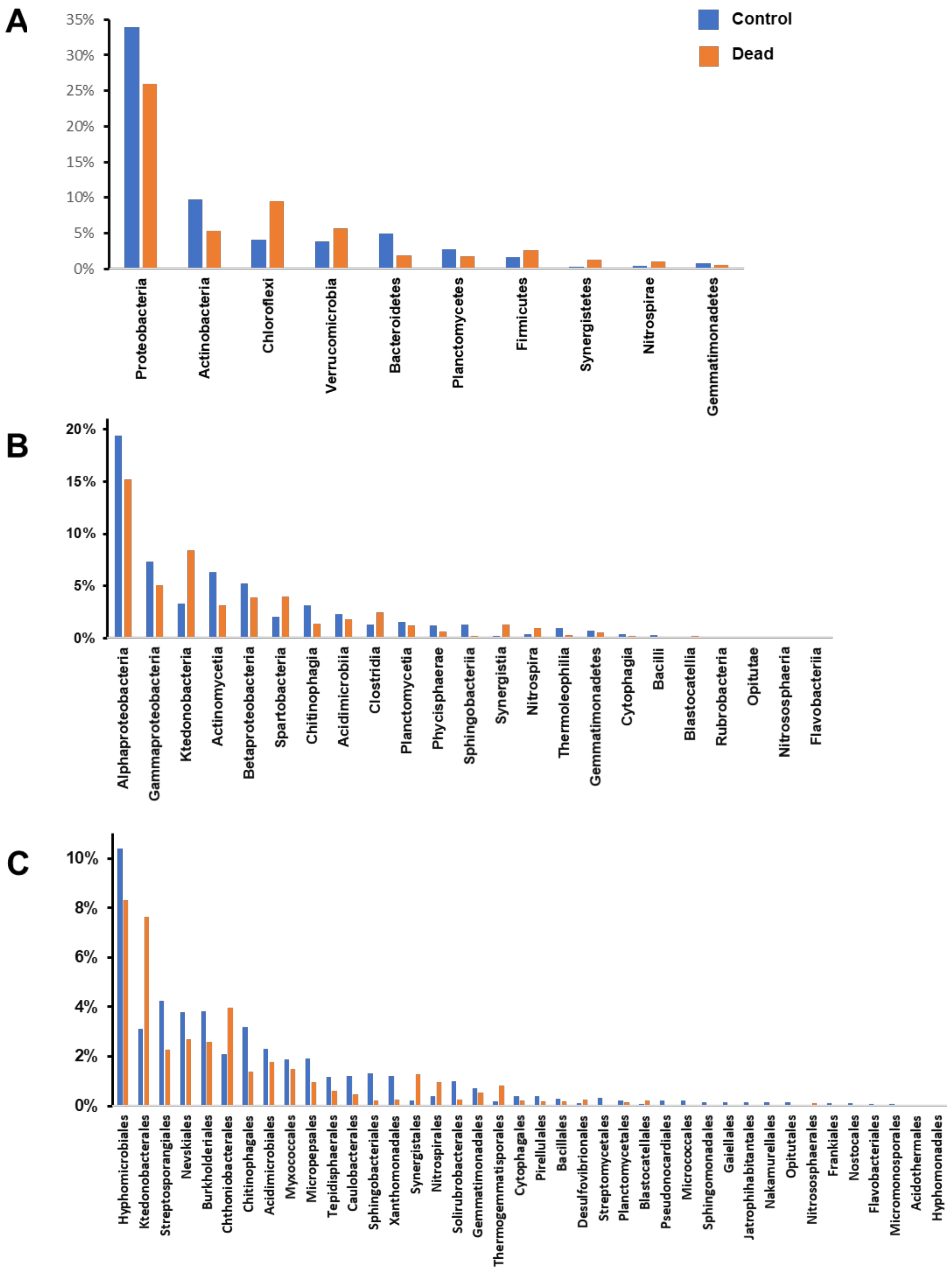

Figure S1. Bar graphs representing the relative abundance of the significantly different ($p < 0.05$) bacterial taxa at the (A) phylum, (B) class, and (C) order levels in the rhizosphere microbiome of healthy and standing dead Korean fir trees.

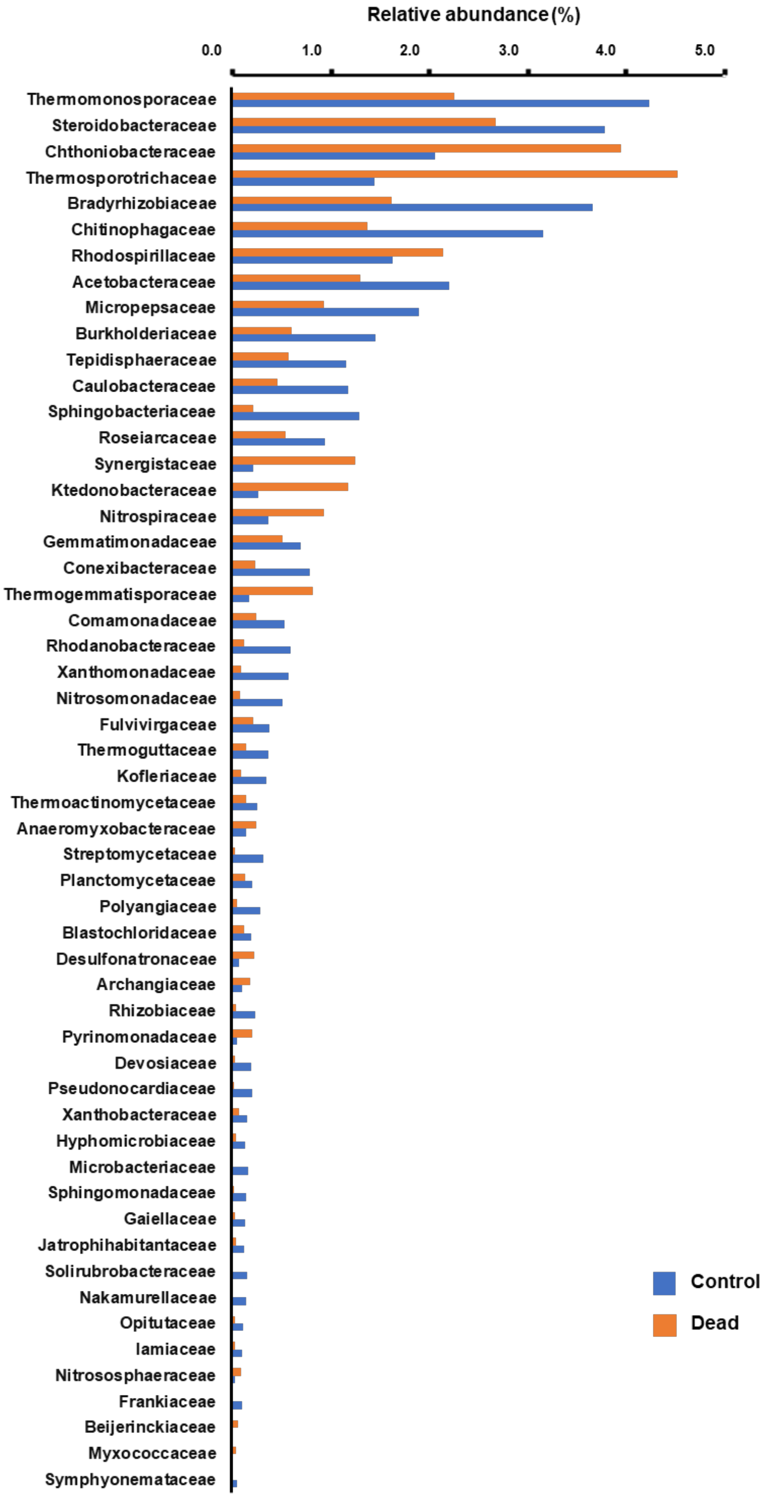

Figure S2. The relative abundance of significantly different bacterial families ($p < 0.05$) between the standing dead Korean fir tree rhizosphere microbiomes and healthy controls. Bars represent the mean values ($n = 15$) of the relative abundance of the bacterial families.
